# Supplementary material for: MetaRibo-Seq measures translation in microbiomes
Source: Nat Commun. 2020 Jun 29;11:3268. doi: 10.1038/s41467-020-17081-z (PMC7324362; doi:10.1038/s41467-020-17081-z)
Supplement: Supplementary file 10 — Supplementary Data 7 [file 41467_2020_17081_MOESM10_ESM.zip › File2/Confidence_High_Taxonomy/127473_out.krona.html]

Javascript must be enabled to view this page.

members
magnitude
magnitudeUnassigned
count
unassigned
taxon
rank

127473\_out

2

2
2
superkingdom

2
phylum
1239

2
class
91061

order
186826
2

family
1300
2

1301
genus
2


SRS143850\_contig\_number\_16849
1
species
1304

species
28037

SRS077022\_contig\_number\_9337
1
